# A Protein E-PilA Fusion Protein Shows Vaccine Potential against Nontypeable Haemophilus influenzae in Mice and Chinchillas

**DOI:** 10.1128/IAI.00345-19

**Published:** 2019-07-23

**Authors:** Carine Ysebaert, Philippe Denoël, Vincent Weynants, Lauren O. Bakaletz, Laura A. Novotny, Fabrice Godfroid, Philippe Hermand

**Affiliations:** a GSK, Rixensart, Belgium; b Department of Pediatrics, The Abigail Wexner Research Institute at Nationwide Children’s Hospital and The Ohio State University College of Medicine, Columbus, Ohio, USA; Washington State University

**Keywords:** chinchilla, *Haemophilus influenzae*, mouse, PilA, protein E, vaccines

## Abstract

PE-PilA is a fusion protein composed of immunologically relevant parts of protein E (PE) and the majority subunit of the type IV pilus (PilA), two major antigens of nontypeable Haemophilus influenzae (NTHi). Here we report on the preclinical evaluation of PE-PilA as a vaccine antigen. The immunogenic potential of the PE and PilA within the fusion was compared with that of isolated PE and PilA antigens. When injected intramuscularly into mice, the immunogenicity of PE within the fusion was equivalent to that of isolated PE, except when it was formulated with alum. In contrast, in our murine models PilA was consistently found to be more immunogenic as a subentity of the PE-PilA fusion protein than when it was injected as an isolated antigen. Following immunization with PE-PilA, anti-PE antibodies demonstrated the same capacity to inhibit the binding of PE to vitronectin as those induced after PE immunization. Likewise, PE-PilA-induced anti-PilA antibodies inhibited the formation of NTHi biofilms and disrupted established biofilms *in vitro*. These experiments support the immunogenic equivalence between fused PE-PilA and isolated PE and PilA. Further, the potential of PE-PilA immunization against NTHi-induced disease was evaluated. After intranasal NTHi challenge, colonization of the murine nasopharynx significantly dropped in animals formerly immunized with PE-PilA, and in chinchillas, signs of otitis media were significantly reduced in animals that had received anti-PE-PilA antibodies. Taken together, our data support the use of PE-PilA as an NTHi vaccine antigen.

## Abstract (precis)

PE-PilA is a fusion protein composed of immunologically relevant parts of protein E (PE) and the majority subunit of the type IV pilus (PilA), two major antigens of nontypeable Haemophilus influenzae (NTHi). Here we report on the preclinical evaluation of PE-PilA as a vaccine antigen. The immunogenic potential of the PE and PilA within the fusion was compared with that of isolated PE and PilA antigens.

## INTRODUCTION

Haemophilus influenzae is a Gram-negative pathogen able to colonize the nasopharynx and to induce disease in both the upper and lower respiratory tracts. Both encapsulated and nonencapsulated (nontypeable H. influenzae [NTHi]) forms of the bacterium exist and exclusively colonize the human host. In adults, NTHi is recognized as the major bacterial cause of exacerbation in chronic obstructive pulmonary disease (COPD) (1, 2). The clinical manifestations are more diverse in children, comprising sinusitis, conjunctivitis, and pneumonia, but NTHi is best known as the predominant pathogen of chronic and recurrent otitis media (OM) (3–6). It can also be considered equivalent to the pneumococcus for its involvement in acute OM (7–12).

Different prophylactic vaccines to target this pathogen were developed more than a decade ago and were evaluated in preclinical studies (13). However, none of them was fully satisfactory, mainly due to sequence variation among the various strains of NTHi. The clinical evaluation of an 11-valent polysaccharide pneumococcal conjugate vaccine using H. influenzae-derived protein D (PD) as the carrier protein showed 35.3% efficacy against NTHi-induced acute OM episodes (14, 15). Although this result opened interesting perspectives for the control of NTHi-induced diseases, it also highlighted the need for additional NTHi vaccine antigens to reinforce this PD-based approach. For that, our strategy has been to combine relevant parts of two well-established vaccine candidates, protein E (PE) and PilA, in a single fusion protein (16).

PE is known as a ubiquitous NTHi adhesive protein important for adhesion to host epithelial cells (17–19). In this regard, the interaction of PE with host laminin plays an important role (20). PE was also shown to bind vitronectin, thereby protecting the bacterium from complement attack (21–23). The host complement system is further dampened by the plasmin that results from the binding of PE to plasminogen (24). PE has recently been proposed to be a relevant NTHi vaccine antigen (25). PilA, the second antigen of the fusion protein, is the major subunit of type IV pili (Tfp) (26). Tfp are hair-like filaments known to be well conserved among H. influenzae isolates (27), binding their target cells through ICAM-1 (28). Tfp are also involved in twitching motility and biofilm formation (29–32). It has been shown that anti-PilA antibodies are able to both prevent the formation of and disrupt established NTHi biofilms (33), which further qualifies PilA as a target vaccine antigen.

In another work, we described and characterized the fusion molecule PE-PilA and demonstrated that the individual structure of each of the two entities is kept within the fusion structure (16). Here, the immunological potential of the fusion molecule is evaluated.

## RESULTS

### Immunogenicity of PE-PilA.

**FIG 1:**
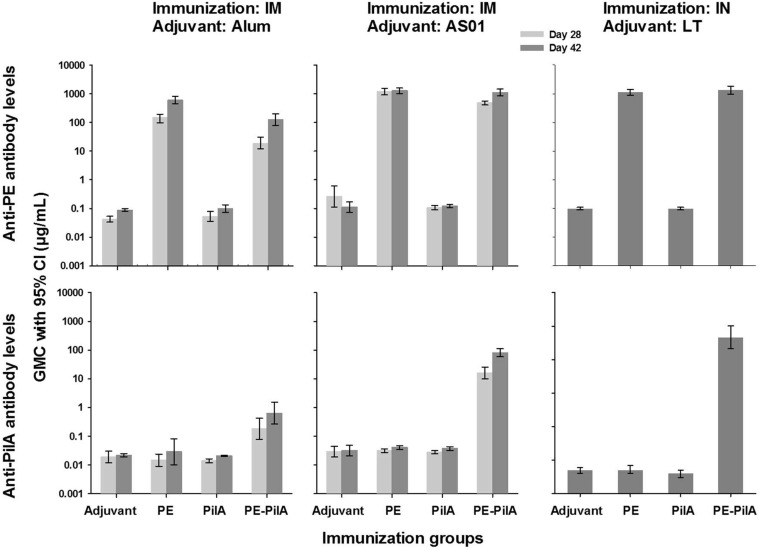
Antibody levels after immunization. Mice (*n* = 33/group) were immunized three times intramuscularly (i.m.) at 2-week intervals with 1 μg PilA, 1 μg PE, or 1 μg PE-PilA adjuvanted with alum or AS01, or mice (*n* = 20/group) were immunized three times intranasally (i.n.) at 2-week intervals with 6 μg PE, 6 μg PilA, or 6 μg PE-PilA adjuvanted with the heat-labile toxin of Escherichia coli (LT). Sera were collected 14 days after the second and the third i.m. injection (days 28 and 42, respectively) or 14 days after the third i.n. injection (day 42). PE-specific and PilA-specific antibody levels were measured by ELISA.

In mice, the humoral responses against PE after PE-PilA immunization were similar to those after immunization with PE alone, as evaluated on day 42 (1,106 μg/ml versus 1,273 μg/ml, respectively, when given intramuscularly [i.m.] and adjuvanted with AS01 and 1,349 μg/ml versus 1,139 μg/ml, respectively, when given intranasally [i.n.] and adjuvanted with the heat-labile toxin [LT] of Escherichia coli). However, this was not the case when the adjuvant was alum (Fig. 1). In the latter case, although 100% of mice seroconverted after immunization with PE-PilA, the level of anti-PE antibodies was almost five times lower than that after immunization with PE alone (126 μg/ml versus 608 μg/ml, respectively).

In mice, PilA was found not to be immunogenic or was very slightly immunogenic when given alone, independently of the adjuvant and the route of immunization (Fig. 1). All anti-PilA levels lay below 0.05 μg/ml. However, PilA within the fusion was found to be more immunogenic than isolated PilA, and this was particularly remarkable when PilA was adjuvanted with AS01 (i.m. administration) or LT (i.n. administration). With alum as the adjuvant, PE-PilA i.m. immunization elicited anti-PilA levels that were 30-fold higher than those elicited by PilA immunization, but when AS01 was used, anti-PilA antibody levels were more than 2,000-fold higher, reaching 80 μg/ml. After i.n. immunization in the presence of LT, the differences were even more striking, since the anti-PilA antibody levels were 463 μg/ml after PE-PilA immunization, which represents more than 10^4^ times the levels obtained after PilA immunization.

### Inhibition of vitronectin binding by PE with antibodies to the PE-PilA fusion.

**FIG 2:**
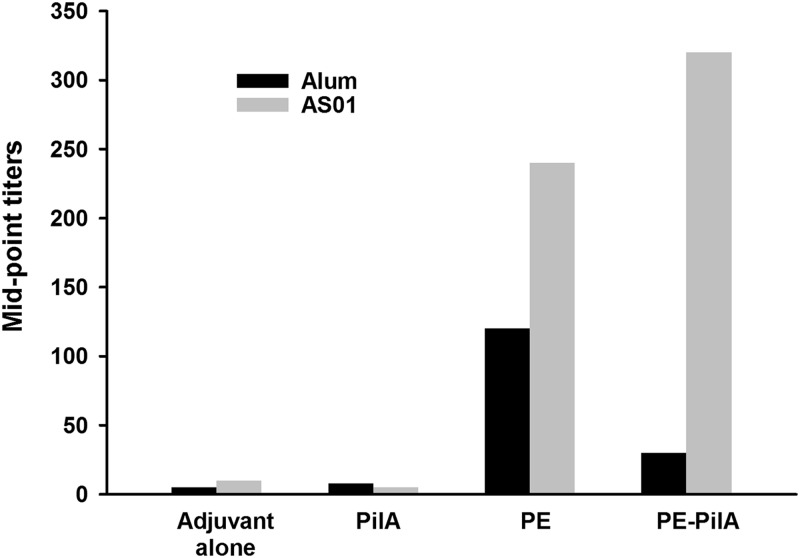
Inhibition of vitronectin binding. Mice (*n* = 33/group) were immunized intramuscularly on days 0, 14, and 28 with 1 μg PE, 1 μg PilA, or 1 μg PE-PilA formulated with alum or AS01. Sera were collected on day 42, and a pool of all sera within each group was made. Twofold dilutions of these serum pools were used to inhibit the binding of vitronectin to PE in microtiter plates. Bound vitronectin was detected by specific antibody. The results are expressed as the dilution of serum able to inhibit 50% of vitronectin binding.

We aimed to determine whether antibodies from mice immunized with PE-PilA were able to inhibit vitronectin binding to PE (Fig. 2). The sera used for the determination of the humoral responses were used for this experiment without adjustment for antibody levels. As was expected, sera from mice immunized with adjuvant alone or PilA alone (negative controls), even if anti-PilA antibodies were generated, were not able to inhibit the binding of vitronectin to PE. When mice were immunized with PE-PilA admixed with alum, the elicited antibodies could inhibit PE-vitronectin recognition, but to a lesser extent than that after immunization with PE admixed with alum, reflecting the difference in anti-PE antibody levels between the two groups. When adjuvanted with AS01, PE and PE-PilA gave comparable anti-PE antibody levels after immunization, and, accordingly, the levels of vitronectin binding inhibition were similar for the two groups.

### Inhibition of biofilm by anti-PE-PilA antibodies.

**FIG 3:**
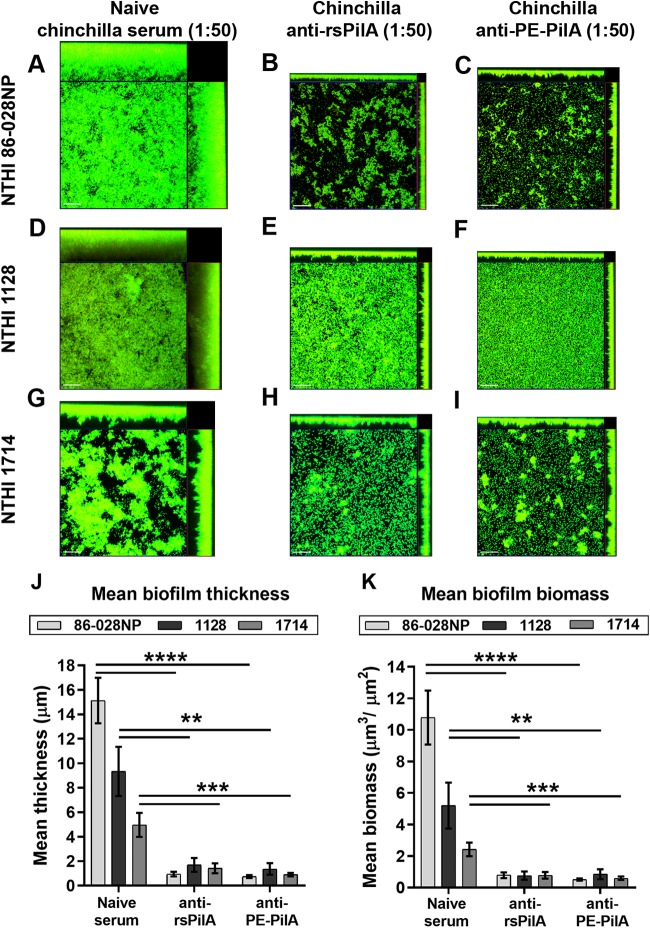
Inhibition of biofilm formation by anti-rsPilA and anti-PE-PilA antibodies. Adult chinchillas (*n* = 6) were immunized with rsPilA or the PE-PilA fusion protein, and sera were collected 10 days after the third immunization and pooled. NTHi biofilms of three different strains (86-028NP, 1714, 1128) were allowed to form in the wells of 8-well chambered glass slides. Immunized or naive (control) serum pools were added to the culture medium during formation of the biofilms. After overnight incubation, biofilms were fixed and then viewed by confocal scanning microscopy. Images were rendered as orthogonal projections to show a top-down view (to depict the relative spatial distribution of the biofilm within the *x-y* planes), as well as a side view (to depict the relative biofilm height within the *z* plane) (A to I), and analyzed with COMSTAT software to evaluate the biofilm thickness (J) and total biomass (K). Bars, 20 μm. *P* values are for immune sera compared with the control. **, *P* < 0.01; ***, *P* < 0.001; ****, *P* < 0.0001.

Type IV pili are composed of a majority subunit, PilA, and play a critical role in biofilm formation, and anti-PilA antibodies are known to play a role in biofilm dispersal. This assumption could be verified in our experiments, in which chinchilla anti-PilA antibodies produced after immunization with PilA were able to inhibit NTHi biofilm formation *in vitro*. Three clinical isolates of NTHi were examined, strain 86-028NP (from which the PilA in the fusion originates) and strains 1714 and 1128 (two strains showing the least PilA amino acid sequence identity to the PilA amino acid sequence of the vaccine antigen) (Table 1). Whereas the biofilms formed by each NTHi strain varied in overall height and architecture, growth in the presence of naive serum did not inhibit the formation of characteristic bacterial towers and intervening water channels (Fig. 3A, D, and G). In contrast, incubation in the presence of anti-PilA antibodies that had been arbitrarily diluted 1:50 prevented the formation of comparable biofilm structures (Fig. 3B, C, E, F, H, and I). Biofilm thickness and biomass were significantly reduced for all three NTHi isolates after incubation with PilA antibodies compared with those after incubation with naive serum (Fig. 3J and K; *P* ≤ 0.01), and anti-PilA or anti-PE-PilA showed comparable efficacy.

**TABLE 1:** NTHi strains used in the study and their characteristics^*a*^

Strain	Tissue	% identity^*b*^	
		PE	PilA
86-028NP	NP	99.3	100
1714	MEF	99.3	88.2
1128	MEF	99.3	81.8
3224A	MEF	99.3	100
3219C	MEF	98.6	88.2

a All strains were from U.S. children with otitis media. NP, nasopharynx; MEF, middle ear fluid.

b Versus the vaccine antigen sequence (using Super Needle software).

**FIG 4:**
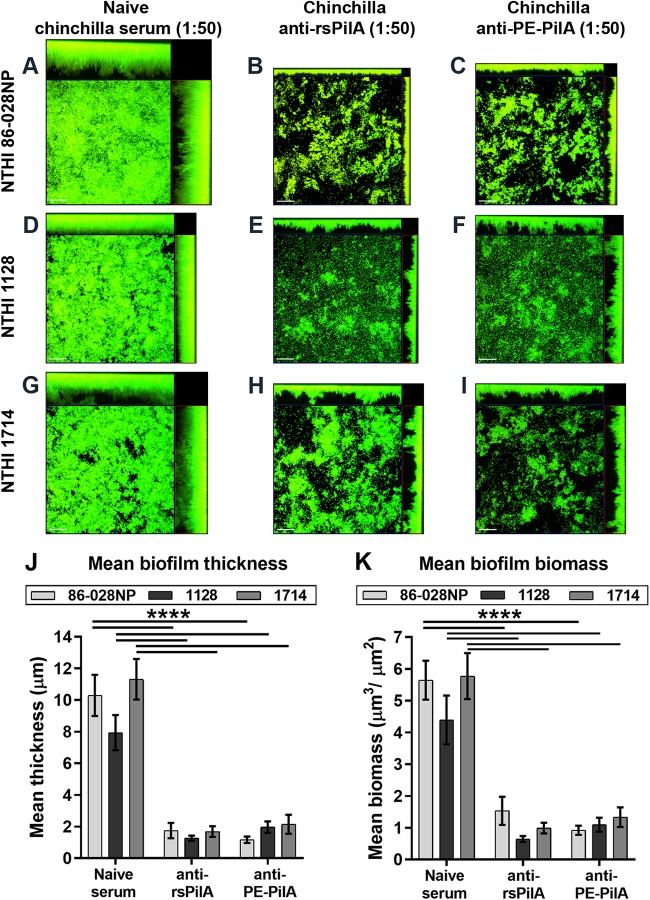
Dispersal of established biofilms by anti-rsPilA and anti-PE-PilA antibodies. Adult chinchillas (*n* = 6) were immunized with rsPilA or the PE-PilA fusion protein, and sera were collected 10 days after the third immunization and pooled. NTHi biofilms of three different strains (86-028NP, 1714, 1128) were allowed to form overnight in the wells of 8-well chambered glass slides. Immunized or naive (control) serum pools were added to the culture medium after the biofilms were established. After overnight incubation, the biofilms were fixed and then viewed by confocal scanning microscopy. Images were rendered as orthogonal projections to show a top-down view (to depict the relative spatial distribution of the biofilm within the *x-y* planes), as well as a side view (to depict the relative biofilm height within the *z* plane) (A to I), and analyzed with COMSTAT software to evaluate biofilm thickness (J) and total biomass (K). Bars, 20 μm. *P* values are for immune sera compared with the control. ****, *P* < 0.0001.

As an additional functional assessment of PilA antibodies *in vitro*, NTHi biofilms were first established for 24 h prior to incubation with naive serum or antiserum against PilA (again, both types of sera were diluted 1:50 prior to use). In contrast to biofilms incubated with naive serum (Fig. 4A, D, and G), treatment of preformed NTHi biofilms with anti-PilA antibodies induced the dispersal of each of the three strains from these structures (Fig. 4B, C, E, F, H, and I). A significant reduction in biofilm thickness and biomass by incubation with anti-PilA and anti-PE-PilA antibodies compared to the biofilm thickness and biomass obtained by incubation with naive serum was observed for each of the three strains (Fig. 4J and K; *P* ≤ 0.0001). As before, anti-PilA and anti-PE-PilA antibodies induced a similar biofilm dispersal efficacy. Collectively, these data demonstrate the ability of anti-PilA antibodies to both prevent the formation and induce the dispersal of biofilms formed by NTHI strains, despite the amino acid sequence diversity within the PilA subunit.

### Protection in the nasopharyngeal colonization model.

To assess the protective activity of PE-PilA vaccination against nasopharyngeal colonization, we used a noninflammatory nasopharyngeal colonization murine model. In this model, the bacteria colonize locally and do not spread to the lungs, due to the small volume of inoculum, and do not infect systemically. Mice were immunized intranasally with adjuvant only (LT), PE alone, PilA alone, or PE-PilA (PE alone, PilA alone, and PE-PilA were all adjuvanted) before they were challenged via the same route with the 3224A or the 3219C NTHi strain (Table 1). The intranasal route was used, as pilot experiments in our laboratories showed no protection with the parenteral route in this model.

**FIG 5:**
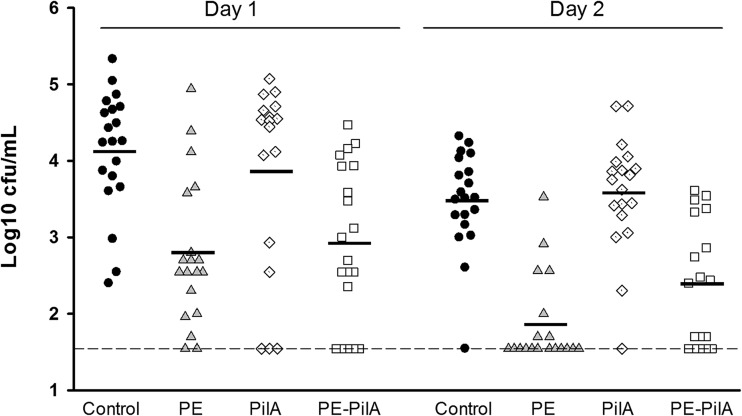
Vaccine efficacy in an NTHi nasopharyngeal colonization model. Mice were immunized intranasally with LT-adjuvanted PE, PilA, or PE-PilA or adjuvant alone (control) before they were intranasally challenged with NTHi strain 3224A. Bacterial colonies in nasal washings were counted on day 1 and day 2 postchallenge and expressed as the mean number of log_10_ CFU. Each symbol represents a mouse. The dashed line indicates the limit of detection; the black horizontal bars are geometric means. Statistical analyses were carried out by two-way ANOVA. Groups were compared to each other over time (Tukey adjusted). *P* was <0.001 for the PE and PE-PilA groups versus the control group, *P* was equal to 0.9937 for the PilA group versus the control group, and *P* was equal to 0.4239 for the PE group versus the PE-PilA group. Representative results of 1 out of 4 experiments with either the 3224A or the 3219C NTHi strain are shown.

When cohorts were compared to each other over time, a significant reduction in the number of bacteria (*P* < 0.001) was shown in the groups immunized with PE-PilA and PE (Fig. 5). No protection was observed with PilA alone (*P* = 0.9937), which is in line with the earlier observation showing that PilA alone is weakly or nonimmunogenic in mice (Fig. 1).

### Protection against otitis media in a chinchilla model.

Antisera generated in adult chinchillas were titrated for anti-PE and anti-PilA antibodies before they were used for passive transfer. As individual antisera, the midpoint titer for anti-PilA was 1,007 and that for anti-PE was 1,661. These titers were within the same log range as those obtained by immunization with PE-PilA fusion (i.e., 1,273 for anti-PilA and 2,984 for anti-PE). Therefore, admixing individual anti-PilA and anti-PE antisera prior to passive transfer yielded immune pools that were comparable in titer to those obtained by immunization with the PE-PilA fusion protein. Two days after intranasal challenge with NTHi, control analysis of the nasopharyngeal lavage fluids indicated that the three groups were equally colonized with the challenge isolate; therefore, any differences in protection observed among the cohorts could be ascribed to the specific antiserum administered.

**FIG 6:**
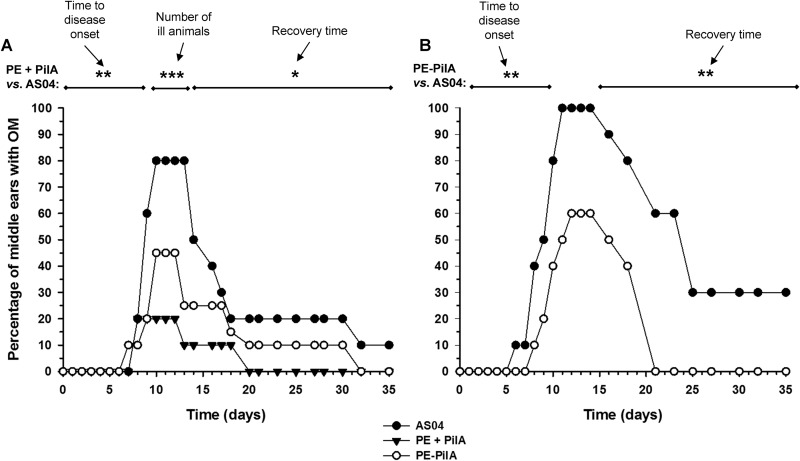
Development of otitis media (OM) in juvenile chinchillas. (A) Adult chinchillas were immunized with adjuvanted PE, PilA, or PE-PilA or adjuvant alone. Sera were collected and used for passive transfer to juvenile chinchillas. Anti-PE and anti-PilA immune sera were combined to form the PE + PilA group. After passive transfer, the juvenile chinchillas were intranasally challenged with NTHi strain 86-028NP, and the development of otitis media was monitored by video otoscopy and tympanometry for 35 days after bacterial challenge. *, *P* < 0.05; **, *P* < 0.01; ***, *P* < 0.001 for PE + PilA versus AS04. (B) Adult chinchillas were immunized with adjuvanted PE-PilA or adjuvant alone. Sera were transferred to juvenile chinchillas, and the animals were intranasally challenged with NTHi strain 86-028NP. The development of otitis media was monitored in a blind manner for 35 days after bacterial challenge. **, *P* < 0.01 for PE-PilA versus AS04.

Transfer of immune serum pools protected to different extents the chinchilla host against experimental otitis media due to the NTHi 86-028NP strain (Fig. 6A). For the four analyzed parameters (e.g., the percentage of middle ears that did not develop disease, the time to disease onset, the time to disease resolution, and the proportion of animals that developed OM), the results for the group that received serum against anti-PE mixed with anti-PilA (PE + PilA) were significantly different from those for the group that received AS04, and only trends were observed for the group that received PE-PilA. Whereas 80% of middle ears showed signs of inflammation in the cohort that received anti-AS04 antibodies, significantly fewer (20%) of the ears had signs of OM after receipt of anti-PilA plus anti-PE antiserum, and while the difference was not statistically significant, 50% of the middle ears in the cohort that received anti-PE-PilA serum remained healthy. The proportion of animals with signs of disease was significantly less in the PE + PilA group than in the AS04 group (*P* < 0.01). Thus, a significantly reduced proportion of middle ears in the PE + PilA cohort compared to the cohort that received anti-AS04 serum had signs of experimental OM (*P* < 0.01). Further, the time to disease onset was significantly longer for the PE + PilA group than for the AS04 group (*P* = 0.005), and whereas a delay was observed in the cohort administered anti-PE-PilA, this outcome was not statistically significant (*P* = 0.07). For those animals in the cohort that received anti-PilA plus anti-PE serum that did develop OM, signs of disease resolved significantly earlier than they did in the control group (*P* = 0.015). The recovery time in the PE-PilA group was not significantly different from that in the control group (*P* = 0.21). When comparing the areas under the curves, as illustrated in Fig. 6, the PE + PilA group was found to be significantly different from the control group (*P* = 0.0293) but not the PE-PilA group (*P* = 0.5893). Vaccine efficacy was determined to be 28.9% (confidence interval [CI], 22.5% to 35.3%) for animals administered anti-PilA plus anti-PE antiserum and 14.7% (CI, 9.7% to 19.7%) for animals administered antiserum against PE-PilA.

In another chinchilla experiment, antisera generated after PE-PilA-AS04 immunization were compared with antisera generated after AS04 injection (Fig. 6B). It was observed that the proportion of animals with signs of otitis media was not different between the two groups (*P* = 0.087). However, the time to disease onset was significantly longer in the PE-PilA group (*P* = 0.0058), and for those animals that developed signs of disease, the recovery time was shorter (*P* = 0.0025) than that in the group receiving anti-AS04 serum. Finally, the area under the curve was lower in the PE-PilA group (*P* = 0.0011). Efficacy for the PE-PilA group was 36.7%. These results show the capacity of PE-PilA immunization to reduce the signs of otitis media.

## DISCUSSION

In a previous work, we designed a fusion protein made of PE and PilA for potential NTHi vaccine applications (16). The aim of the present work was to evaluate the immunogenicity of this fusion protein and the functionality of the elicited antibodies.

PE was found to be immunogenic, producing high levels of antibodies in BALB/c mice when administered i.m. or i.n., and after immunization with PE-PilA, anti-PE antibodies were also generated to a level similar to that achieved with PE alone, except when formulated with alum, which remains an unexplained observation. In contrast, PilA was found not to be immunogenic in BALB/c mice when administered as an isolated protein. This was surprising, as PilA demonstrated immunogenicity in other animal models, such as chinchillas and rabbits (27, 33). Being incorporated within PE-PilA rendered PilA more immunogenic in these mice, and this was particularly evident when formulated with AS01 for i.m. injections or when given intranasally in the presence of LT.

Both the anti-PE and the anti-PilA antibodies generated after immunization with PE-PilA were functional. Anti-PE antibodies were able to inhibit the binding of PE to vitronectin. Knowing that the binding of PE to vitronectin can protect the bacterium from complement attack (21, 22), the presence of inhibiting antibodies may render the bacterium more vulnerable to that killing mechanism. Likewise, the anti-PilA antibodies generated by PE-PilA immunization were functional, as anti-PilA antibodies were shown to prevent biofilm formation. The mechanism in play involves the inhibition of Tfp-mediated adherence and the blockade of NTHi twitching motility, which are foundational steps to initiate, organize, and develop these structures (26, 29, 31–33). Further, the elicited anti-PilA antibodies were able to induce the disruption of established biofilms, which is achieved via a top-down dispersal event that is dependent on PilA expression and LuxS-mediated quorum signaling (33, 34). This capacity to impede biofilm formation and to disperse already formed biofilms is an important feature in the fight against the pathogen. Indeed, biofilms have been described in children suffering from otitis media with effusion or recurrent acute OM (35–38), and NTHi is a predominant organism associated with OM. In addition, disruption of biofilms releases the bacteria in the planktonic state, in which they are more vulnerable to antibodies and antibiotics (34, 39–41).

After the demonstration of its ability to elicit functional antibodies *in vitro*, we aimed to determine whether PE-PilA was able to induce protection in animal challenge models. Our results showed that immunization with PE-PilA was able to prevent NTHi colonization in the mouse nasopharynx, similar to immunization with PE alone, which was demonstrated for two different NTHi strains. This model is noninflammatory and does not engage the innate side of immunity (unpublished information), findings which highlight the potential role of the vaccine-induced antibodies in preventing colonization. In addition to the NTHi colonization model in mice, we used the chinchilla model to study the ability of passively transferred anti-PE-PilA antibodies to prevent the occurrence of otitis media. The chinchilla is a well-established and reliable model for otitis media (42). Biofilms form in the middle ear of the chinchilla after NTHi challenge (35), and *in vivo*-formed biofilms are known to contain large amounts of type IV pilin protein (43), making them the targets of choice for anti-PilA antibodies. In earlier chinchilla experiments, anti-PilA antibodies alone were shown to reduce signs of otitis media symptoms by 42%, whereas the reduction was only 21% for anti-PE antibodies (unpublished results). In the present study, anti-PE-PilA antibodies were as efficient as anti-PilA antibodies at impacting biofilms *in vitro*. Therefore, it was particularly interesting to study the behavior of the PE-PilA-induced antibodies in the chinchilla model. However, the outcome was less striking in chinchillas than *in vitro*, although it could be concluded from the two chinchilla experiments that PE-PilA-induced antibodies are able to reduce the signs of otitis media. Some discrepancies between *in vitro* and *in vivo* experiments and between different animal models are not uncommon and may rely on the intrinsic specificities of the respective animals’ immune systems.

In conclusion, PE-PilA has shown potential as a vaccine antigen, more particularly based on the capacity of PE-PilA-induced antibodies to impede NTHi nasopharyngeal colonization, to both prevent and disrupt bacterial biofilms, and to inhibit PE-vitronectin binding. Based on these and other results, the development of this vaccine antigen has been pursued. It has been decided to associate it with PD, another NTHi antigen, and this combination has now reached the clinical development phase (44).

## MATERIALS AND METHODS

### Antigens and adjuvant systems.

Recombinant PE antigen consisted of amino acids 22 to 160 of the PE sequence completed by a His tag. Its size was approximately 18 kDa. The initial gene fragment originated from the NTHi 772 strain. Periplasmic expression was ensured by the PelB signal sequence upstream of the PE sequence. The construct was expressed in Escherichia coli BLR(DE3) cells, and the resulting antigen was purified by immobilized metal affinity chromatography.

The recombinant isolated PilA antigen consisted of amino acids 40 to 149 of the PilA sequence plus 17 amino acids from PilB. The initial gene fragment originated from the NTHi 86-028NP strain and was associated with a sequence for the His tag. The construct was expressed in E. coli Origami B(DE3) cells, and the resulting antigen was purified by immobilized metal affinity chromatography. The His tag was removed by use of a thrombin cleavage capture kit (Novagen), yielding an antigen of approximately 12 kDa. In the biofilm dispersal experiments, antibodies directed against another recombinant PilA antigen, called rsPilA (described in reference 27), were used.

PE-PilA is a 28.8-kDa His-tagged fusion protein encompassing amino acids 19 to 160 of PE and amino acids 40 to 149 of PilA linked by a GG amino acid linker (16). The gene construct was expressed in E. coli BLR(DE3), and the secreted peptide was purified from the cell lysate through several chromatography steps.

AS01 is a liposome-based adjuvant system containing 3-*O*-desacyl-4′-monophosphoryl lipid A (MPL) and QS-21 (Quillaja saponaria Molina, fraction 21; licensed by GSK from Antigenics LLC, a wholly owned subsidiary of Agenus Inc., a Delaware, USA, corporation) (45). AS04 is an adjuvant system composed of MPL adsorbed on an aluminum salt (46).

### NTHi strains.

Different strains of NTHi were used in the study. They are detailed in Table 1.

### Animals.

The female BALB/c Ola Hsd mice used in this study were purchased from Harlan (Horst, The Netherlands). They were 5 weeks old at the time of the first immunization. All mouse studies were ethically reviewed (GSK, Belgian site, Ethical Committee protocols 04/88/02A, 04/88/03A, and 07/136/02A) and carried out at GSK (Rixensart, Belgium) in accordance with European Directive 2010/63/EU and the GSK policy on the care, welfare, and treatment of animals. Upon arrival, the mice were acclimated for at least 5 days. Afterwards, they were randomly allocated to the different groups. All animals had free access to food and 0.22-μm-pore-size-filtered tap water. Nesting material was provided with nonstructural enrichment material. The air supplied in the housing room was 100% fresh air filtered by an EPA filter, and the ventilation was at least 20 cycles per hour. The animal room conditions were set as follows: temperature, 20 to 24°C; humidity, 55% (range, 40% to 65%); and light-dark cycle, 12 h-12 h.

All chinchilla studies were conducted at The Abigail Wexner Research Institute at Nationwide Children’s Hospital (Columbus, OH, USA). Prior to enrollment, juvenile chinchillas (Chinchilla lanigera; from Rauscher’s Chinchilla Ranch, LLC, LaRue, OH, USA) were nominally bled, and individual serum samples were assayed by Western blotting to confirm that no animal had a significant preexisting level of antibody against any outer membrane protein of NTHi. The animals were then clustered into cohorts of 9 or 10 animals each on the basis of body weight (average weight per cohort = 411 g).

Adult chinchillas (from Rauscher’s Chinchilla Ranch, LLC, LaRue, OH, USA) weighed 625 ± 23 g at the study start. All procedures with the chinchillas were performed in compliance with morbidity/exclusion criteria detailed in a protocol approved by The Abigail Wexner Research Institute at Nationwide Children’s Hospital Animal Care and Use Committee (number 01304AR) and in compliance with the United States Department of Health and Human Services *Guide for the Care and Use of Laboratory Animals* (47).

### Immunization of mice.

Groups of 33 mice were immunized i.m. on days 0, 14, and 28 with 1 μg PilA, 1 μg PE, or 1 μg PE-PilA adjuvanted with alum or AS01. Control injections consisted of alum or AS01 alone. The IgG levels directed against each antigen were determined by enzyme-linked immunosorbent assay (ELISA) of sera collected on days 28 (day 14 after the second injection) and 42 (day 14 after the third injection).

### ELISA.

To determine anti-PE or anti-PilA IgG levels, microtiter plates were coated with PE or PilA (2 μg/ml or 4 μg/ml, respectively, in carbonate buffer) overnight at 4°C. After washing, serial 2-fold dilutions of murine sera (starting at 1/500 for the PE assay or 1/20 to 1/500 for the PilA assay, depending on the experiment, in phosphate-buffered saline [PBS] containing 0.05% Tween 20 [PBS-T] for 1 h at 25°C).

Afterwards, in both assays, peroxidase-conjugated goat anti-mouse IgG antibodies (1/2,500 or 1/1,250 in PBS-Tween; code 115-035-003; Jackson Laboratories) were added for 1 h at 25°C. The colorimetric reaction was obtained by the addition of *o*-phenylenediamine dihydrochloride in citrate buffer in the presence of hydrogen peroxide for 15 min and stopped by the addition of 1 N HCl. The plates were read in a spectrophotometer at 490 and 620 nm. In both cases, an in-house-calibrated reference serum sample was used, and IgG concentrations (expressed in micrograms per milliliter) were calculated by the 4-parameter method using Soft Max Pro software.

### Inhibition of vitronectin binding.

The sera collected on day 42 for the determination of humoral responses were also used for the inhibition of the vitronectin binding assay, which is a method to assess the functionality of anti-PE antibodies. A pool of all serum samples within each group was made. The vitronectin binding assay was carried out in microtiter plates. The plates were coated with PE (5 μg/ml in PBS) for 2 h at 37°C. After washing, saturation of the nonspecific binding sites was done by incubation with PBS–1% bovine serum albumin (BSA), and then 2-fold serial dilutions of heat-inactivated immune murine sera (in PBS-T–0.02% BSA) were added to the wells for overnight incubation at 4°C. After washing, vitronectin (4 μg/ml; catalog number SRP3186; Sigma-Aldrich) was added and the plates were incubated for 1 h at 37°C. Finally, after another washing step, bound vitronectin was detected by the addition of horseradish peroxidase-conjugated sheep antivitronectin antibodies (1/1,000 in PBS-T for 30 min at 37°C; catalog number L12050350 C12120412; U.S. Biological), followed by *o*-phenylenediamine dihydrochloride as described in the previous paragraph. The midpoint titer (corresponding to the first dilution of murine sera able to inhibit 50% of binding) of each tested pool was determined.

### Effects of antibodies on biofilm.

Biofilm experiments were carried out with three different strains of NTHi: the homologous strain 86-028NP and the two heterologous strains 1714 and 1128, the PilA proteins of which show 88.2 and 81.8% amino acid sequence identity with PilA of the vaccine antigen, respectively (Table 1).

### Dispersal of established biofilm.

To obtain biofilms, as previously described (48), overnight cultures of NTHi were resuspended in 5 ml equilibrated (37°C, 5% CO_2_) brain heart infusion broth supplemented with 2 μg NAD/ml and 2 μg heme/ml (sBHI) so that the optical density at 490 nm was 0.65. The bacteria were diluted 1:6 in equilibrated sBHI in a 50-ml nonclosed sterile conical tube and incubated at 37°C in 5% CO_2_. After 3 h, bacteria were diluted 1:2,500 in equilibrated sBHI, and 200 μl of the bacterial suspension was added to each well of an 8-well chambered glass slide (catalog number 155411; Nunc Lab-Tek) for an overnight incubation at 37°C in 5% CO_2_, during which biofilms formed. Medium was refreshed for an additional 8 h of incubation, after which immunized or naive serum pools (serum pools from the adult chinchillas used in the *in vivo* study diluted 1:50 in sBHI; see below) were added to the chambers. After 16 h at 37°C in 5% CO_2_, biofilms were washed with sterile saline, incubated with a Live/Dead BacLight bacterial viability kit (catalog number L7007; Invitrogen), and fixed for 1 h at room temperature with 10% formalin. Biofilms were immediately viewed on a Zeiss 510 confocal laser scanning microscope, and images were analyzed with COMSTAT software (49).

### Inhibition of biofilm formation.

The beginning of the procedure for the inhibition of biofilm formation was the same as that described in “Dispersal of established biofilm” above. After the bacteria were added to each well of an 8-well chambered glass slide, incubation took place for 1 h. Then, medium was replaced by immune or naive sera (1:50 in sBHI) for 4 h at 37°C in 5% CO_2_. The chambers were rinsed, and biofilms were allowed to form overnight or longer (the medium was refreshed every 16 h in such a case) in sBHI. Biofilms were stained for viability, fixed, and then viewed by confocal microscopy, and images were analyzed with COMSTAT software.

### Nasopharyngeal colonization model.

For the nasopharyngeal colonization model, groups of 20 mice were immunized i.n. (10 μl in one nostril) on days 0, 14, and 28 with 6 μg PE, PilA, or PE-PilA adjuvanted with LT (50 μg/ml; except at the third immunization). The IgG levels against each antigen in sera collected on day 42 (day 14 after the third injection) were measured by ELISA. The animals were also challenged i.n. on day 42 with 5 × 10^6^ CFU (the number of CFU of the 3224A or 3219C NTHi strain in 10 μl in one nostril). Full nasal cavities were dissected 1 and 2 days after the challenge and homogenized, and the resulting suspension was cultured overnight at 37°C on chocolate agar to determine the bacterial load.

### Chinchilla model.

Cohorts of six adult chinchillas were subcutaneously immunized three times at 28- to 30-day intervals with 10 μg of PE, PilA, or PE-PilA, all of which were adjuvanted with AS04. The control group received AS04 alone. Animals were bled 10 days after receiving the third immunization, and the sera were pooled by cohort before passive transfer to the juvenile chinchillas.

The passive transfer study was carried out as follows: at 7 days before NTHi challenge, an adenovirus serotype 1 strain was intranasally inoculated (2 × 10^7^ 50% tissue culture infective doses/ml in 200 μl, 100 μl per naris) to the young chinchillas (9 or 10/group). At 1 day before the challenge, the chinchillas were intracardially injected with (mixes of) adult chinchilla antibodies obtained via immunization with the different proteins (5 ml serum/kg of body weight). One group received a pool of serum from animals immunized with PE mixed 1:1 with serum from animals immunized with PilA (the PE + PilA group). Another group received a pool of serum from animals immunized with PE-PilA (the PE-PilA group). A third group received serum from adult animals immunized with AS04 alone (AS04 group). On the day after that, the young chinchillas were intranasally challenged with 10^8^ CFU of NTHi (100 μl per naris). Colonization status was verified 2 days later by nasopharyngeal lavage and culture of the lavage fluids on chocolate agar supplemented with 15 μg ampicillin/ml to limit the growth of the nasopharyngeal flora. Further, the general health of the juvenile chinchillas was evaluated, and the occurrence of ear infection was monitored by video otoscopy and tympanometry for 35 days after the bacterial challenge (50).

### Statistical analyses.

Inhibition of vitronectin binding was analyzed by analysis of variance (ANOVA), followed by Tukey’s adjusted test. Vaccine efficacy in the nasopharyngeal model was measured by two-way ANOVA with groups and time as factors. Groups were compared to each other by Tukey’s adjusted test. Biofilm thickness and biomass were analyzed by one-way ANOVA. For the passive transfer experiments, a sample size of 10 allowed detection of a 65% difference between two proportions (OM incidences) with a power of 80%, using a χ^2^ test. Four different statistical analyses were performed in each experiment to compare the vaccinated groups and the adjuvant control group for the proportion of animals developing OM (Fisher’s exact test), the time to OM onset (first day of disease), the recovery time (last day of disease), and the area under the score curve. Results were considered significant when *P* values were equal to or below 0.05.
